# Excessive Daytime Sleepiness and Body Composition: A Population-Based Study of Adults

**DOI:** 10.1371/journal.pone.0112238

**Published:** 2014-11-10

**Authors:** Amie C. Hayley, Lana J. Williams, Gerard A. Kennedy, Michael Berk, Sharon L. Brennan, Julie A. Pasco

**Affiliations:** 1 IMPACT SRC, School of Medicine, Deakin University, Geelong, Australia; 2 Institute for Breathing and Sleep, Austin Health, Melbourne, Australia; 3 Department of Psychiatry, The University of Melbourne, Parkville, Australia; 4 School of Psychology, Counselling & Psychotherapy, Cairnmillar Institute, Camberwell, Australia; 5 NorthWest Academic Centre, Department of Medicine, The University of Melbourne, St Albans, Australia; 6 Orygen Research Centre, Parkville, Australia; 7 Florey Institute for Neuroscience and Mental Health, Parkville, Australia; 8 Australian Institute for Musculoskeletal Science, North West Academic Centre, Department of Medicine, The University of Melbourne, St Albans, Australia; University of Pennsylvania School of Medicine, United States of America

## Abstract

**Background:**

Excessive daytime sleepiness (EDS) is often associated with increased adiposity, particularly when assessed in the context of samples of sleep-disordered patients; however, it is unclear if this relationship is sustained among non-clinical, population-based cohorts. This study aimed to investigate the relationship between EDS and a number of body composition markers among a population-based sample of men and women.

**Methods:**

This study assessed 1066 women aged 21–94 yr (median = 51 yr, IQR 35–66), and 911 men aged 24–92 yr (median = 60 yr, IQR 46–73) who participated in the Geelong Osteoporosis Study (GOS) between the years 2001 and 2008. Total body fat mass was determined from whole body dual-energy X-ray absorptiometry scans, and anthropometric parameters (weight, height, and waist circumference) were measured. Lifestyle and health information was collected via self-report. Sleepiness was assessed using the Epworth Sleepiness Scale (ESS). Scores of ≥10 were considered indicative of EDS.

**Results:**

*Women:* After adjusting for age, alcohol intake, antidepressant medication use and physical activity, EDS was associated with greater waist circumference and body mass index (BMI). EDS was also associated with 1.5–1.6-fold increased odds of being overweight or obese. *Men:* After adjusting for age, alcohol use, physical activity and smoking status, EDS was associated with greater BMI. These findings were not explained by the use of sedative or antidepressant medication. EDS was also associated with 1.5-fold increased likelihood of being obese, independent of these factors. No differences in lean mass, %body fat, or %lean mass were detected between those with and without EDS for men or women.

**Conclusions:**

These data suggest that EDS is associated with several anthropometric adiposity profiles, independent of associated lifestyle and health factors. Among women, symptoms of EDS are pervasive at both overweight and obese BMI classifications; suggesting a need for further clinical examination to assess possible temporal associations with underlying sleep pathology.

## Introduction

Prevalence of overweight and obesity are increasing significantly throughout both the developed [1] and developing worlds [2]. Recent local studies report that between 3.5–7.6% of children and approximately 20% of adults are classified as obese (kg/m^2^) [3]–[5]. This trend is consistently mirrored in most countries, across socioeconomic standings [2] and gender [6]. The clinical and societal implications of obesity have been well documented. Excess weight and body fat, particularly that which is viscerally or centrally located, has been found to be a predisposing factor for an increased risk for type 2 diabetes, poorer cardiovascular outcomes, stroke, heart attacks, increased medical comorbidity, depression and increased risk of sleep disorders [7], [8].

Obesity has been proposed to affect sleep architecture via a combination of impairment to physiological mechanisms, which act to maintain upper airway patency, and functional alterations between respiratory drive and load compensatory mechanisms [9]. Consequently, obese individuals are more likely to exhibit decreased respiratory function, resulting in periods of hypoxemia, hypercapnia and respiratory resistance [10], [11]. Nocturnal respiratory disturbances observed in obese individuals often manifest as instances of compromised sleep architecture and excessive daytime sleepiness (EDS) [12]. Indeed, obese individuals are more likely to report symptoms of EDS than are non-obese individual [13], and this association has also been found among non-apnoeic obese populations [12]. Although the relationship between OSA and obesity has been well documented, particularly among clinical samples [14], several authors have described a lack of association between these factors, particularly at a population level [14], [15]; and have instead cited peripheral indicators such as metabolic syndrome and lifestyle factors as having a closer association with EDS [16].

The role of lifestyle and health factors as contributing factors in the association between EDS and body composition is unclear. Longitudinal research has shown that engagement in regular exercise aids in the reduction of total body fat, %body fat, BMI and weight and improves sleep quality especially slow wave sleep [17], [18], and dietary improvements contribute to a greater reduction of body weight and abdominal fat [19]. Conversely, poor diet, maladaptive lifestyle habits, and physical inactivity have all been associated with greater index of adiposity and obesity [20], as well as poorer sleep quality [21]. Despite these findings, there is a paucity of studies incorporating these factors as possible contributory factors when assessing EDS and body composition. Indeed, many of these studies investigate aspects of lifestyle and health in isolation, or among specific patient groups [19], and thus it is unclear whether these factors represent possible modifiable factors.

The mechanism by which EDS influences body composition requires further elucidation; particularly in population-based samples. Similarly, the role of a number of lifestyle and health variables as contributory factors to this association is equivocal. Daytime sleepiness is a common symptom among obese people, and lifestyle and health factors have been shown to represent possible modifiable factors in the expression of both conditions. Therefore, there is a need to assess the relationship between EDS and body composition in a representative, population-based cohort, whilst controlling for a number of commonly associated lifestyle and health factors.

## Methods

### Participants

This study examined men and women who participated in the Geelong Osteoporosis Study (GOS). The GOS is a large, population based, age-stratified cohort study conducted in south-eastern Australia. Participants were selected at random from Commonwealth electoral rolls for the Barwon Statistical Division.

Between the years 1993–1997, 1494 women were randomly recruited, representing 77.1% participation [22]. At the 10-year follow up (2004–2008), 881 women from the original sample returned (82.1%) and were complemented by the inclusion of an additional 246 randomly-selected women aged between 20–29 years. Of the 1126 women who participated in the 10-year follow up, participants for whom weight and/or height (n = 38) and sleep data (n = 22) were not available, were excluded, resulting in a total of 1066 eligible women aged 20–94 years.

Between the years 2001–2006, 1540 men were recruited for baseline analysis, representing a response of 67% [22]. Of the 979 men who participated in the 5-year follow up (81% response), participants for whom follow-up (n = 1) weight and/or height (n = 35) and sleep data (n = 32) were not available were excluded from analysis, resulting in a total of 911 eligible men aged between 24–92 years.

Written informed consent was obtained from each participant. Approval to conduct this study was granted under the project numbers 92/01 and 00/56 from the Barwon Health Human Research Ethics Committee.

### Measurements

#### Epworth Sleepiness Scale

Instances of EDS were identified using the Epworth Sleepiness Scale (ESS). Detailed descriptions of the psychometric properties of the ESS have been detailed elsewhere [23]. The ESS is considered to have good internal validity and retest reliability, and is considered a low-cost and effective measure of assessing sleepiness in adults [24]. The ESS assesses an individual’s sleep propensity and likelihood of dozing off in both soporific and engaging tasks via a self-administered 8-item scale [24]. Sleepiness is assessed using a 4-point Likert scale, referring to an individuals’ likelihood of dozing in that particular situation (0 = would never doze, 1 = slight chance of dozing, 2 = moderate chance of dozing, 3 = high chance of dozing). Scores range from 0–24, with higher scores reflecting higher levels of sleepiness. At present, there are no universally adapted cut-off ranges for the ESS; however similar studies have utilized a cut-off score of ≥10 to indicate EDS [25].

#### Body composition

Anthropometric measurements were recorded objectively. Height and weight were measured to the nearest ±0.1 cm and ±0.1 kg, respectively. Body mass index (BMI) was calculated as weight/height squared (kg/m^2^). A BMI of <25 kg/m^2^ was considered normal weight, ≥25 kg/m^2^ to <30 kg/m^2^ was considered overweight, and ≥30 kg/m^2^ as obese. Waist circumference was measured halfway between the margin of the lower rib and iliac crest using a narrow metal anthropometric tape measure. Participants were classified as obese if they reported a waist measurement of ≥102 cm (men) or ≥88 cm (women) [26]. Fat mass was determined from whole body dual-energy X-ray absorptiometry scans. Total percentage fat mass, lean mass and bone mineral content (BMC) were calculated by dividing fat mass, lean mass or BMC by the sum of fat mass, lean mass and BMC (expressed as %). Automated upper arm digital blood pressure monitors (UA-767) were used to measure systolic and diastolic blood pressures (mmHg).

#### Lifestyle and heath factors

Information regarding alcohol consumption and daily energy intake were obtained from the Cancer Council food frequency questionnaire [27]. Daily alcohol usage was expressed as gram intake per day (g/day), and energy intake was assessed as kilojoule intake per day (kJ/day). Physical activity levels were assessed via self-report and transformed into a binary variable. Participants were classified as ‘active’ if they reported ‘moving, walking and working energetically and participating in vigorous exercise’. Alternatively, participants were classified as sedentary. Self-reported tobacco smoking was documented, and grouped as ‘current’ or ‘not’. Medication use (sedatives and antidepressants) was determined using self-report and usage was classified as ‘current’ if participants reported use at the time of assessment. Socioeconomic Status was determined by use of the Socio-economic Index for Areas (SEIFA) index values ascertained from the 2006 Australian Bureau of Statistics data. SEIFA values were applied to obtain an aggregated Index of Relative Socio-Economic Advantage and Disadvantage (IRSAD). Participants were categorised into five groups, according to quintiles of IRSAD for the study region. Quintile 1 represented the most disadvantaged group, and quintile 5 represented the most advantaged. Participants’ perceived general health status was obtained via self-report and classified on a 5-point Likert scale, expressed as (1) Excellent, (2) Very good), (3), Good, (4), Fair, and (5) Poor. The presence of diabetes was identified by combination of self-report and/or use of insulin or oral hypoglycemic agents.

### Statistical analysis

Differences in characteristics between those with and without EDS were analysed using t-tests for parametric continuous data, Mann-Whitney test for non-parametric continuous variables and Chi-Square test analysis for discrete variables. Fisher’s Exact Test was used for non-parametric variables where expected cell sizes were less than five. Differences in body composition (those expressed as continuous variables) between those with and without EDS were compared using linear regression techniques. Differences in body composition expressed as categorical variables (waist circumference, BMI categories) were tested using logistic regression models. In all models, EDS (yes/no) was applied as the exposure variable. Age, alcohol use, physical activity levels, smoking status, energy intake, sedative and antidepressant use were tested sequentially, and potential confounders and effect modifiers were checked in statistical models. All statistical analyses were completed using Minitab (Version 16; Minitab, State College Pa).

## Results

### Women

Subject characteristics are presented in Table 1. Overall, 146 (13.7%) women were classified as having EDS. The median age for this sample was 51 yr (range 20.9–93.6 yr), and women with EDS tended to be slightly older than those who did not report EDS. Median BMI for this cohort was 26.3 kg/m^2^. A large percentage (60.0%) of this sample was considered overweight (31.1%) or obese (28.9%) based on BMI criteria.

**Table 1:** Characteristics of men and women, with and without EDS*.

	Women				Men			
	All	No	Yes*	*p*	All	No	Yes*	*P*
	N = 1066	n = 920	n = 146		n = 911	n = 789	n = 122	
*Body* *composition*								
Age (years)	51.0(34.6–65.6)	50.8(34.4–65.9)	51.6(38.5–64.3)	0.44	59.6(45.9–72.8	58.7(44.9–71.6)	66.3(52.3–80.4)	**<0.01**
Height (cm)	162. 1(157.8–167.0)	162.0(157.8–167.0)	162.9(157.0–166.9)	0.80	174.9(170.1–179.6)	175.1(170.6–179.7)	173.1(168.0–178.5)	**<0.01**
Weight (kg)	69.3(61.4–80.8)	68.6(61.0–80.2)	73.6(63.1–83.0)	**<0.01**	82.8(74.6–92.8)	82.6(74.7–93.0)	83.4(72.9–94.1)	0.74
BMI (kg/m^2^)	26.3(23.4–30.9)	26.1(23.3–30.5)	27.9(24.4–32.7)	**<0.01**	27.2(24.7–29.6)	27.0(24.7–29.6)	27.9(25.3–30.4)	**0.06**
BMI groups^1^				**<0.01**				0.24
Normal	427(40.1%)	383(41.6%)	44(30.1%)		248(27.3%)	220(27.9%)	28 (23.0%)	
Overweight	639(60.0%)	537(54.4%)	102(69.7%)		458(50.3%)	398(50.5%)	60 (49.2%)	
Obese	308(28.9%)	252(27.4%)	56(38.4%)		204(22.4%)	170(21.6%)	34 (27.9%)	
Waist circumferencetotal (cm)	86.0 (77.0–97.0)	85.0(76.6–97.0)	89.0(81.5–100.0)	**<0.01**	97.8(90.2–105.0)	97.0(90.0–105.0)	100.0(91.0–108.3)	**0.03**
Waist circumference(obese)								
≥102 cm (men)	–	–	–		318(35.0%)	264(33.5%)	54 (44.3%)	**0.02**
≥88 cm (women)	483(46.2%)	404(44.9%)	79(54.5%)	**0.03**	–	–	–	–
Lean mass (g)	39275(36364–42555)	39188(36215–42504)	39831(37154–42958)	0.09	57138(52663–62350)	57186(52821–62339)	56093(51872–62398)	0.42
% lean mass	56.7(51.6–62.8)	57.1(51.8–62.9)	55.2(51.1–61.6)	0.09	68.4(64.4–72.8)	69.0(64.5–73.0)	67.4(64.1–72.3)	0.15
Fat mass (g)	26759(20140–35686)	26172(19900–35337)	29752(22676–37725)	**0.03**	22962(18008–28688)	22667(18010–28448)	24518(17825–29777)	0.16
% body fat	39.6(33.2–45.0)	39.1(33.1–45.0)	41.1(34.8–45.7)	0.12	27.6(23.2–32.1)	27.4(23.1–32.0)	29.1(23.9–32.5)	0.11
% BMC	3.7(3.4–4.2)	3.7(3.4–4.2)	3.7(3.4–4.1)	0.45	3.8(3.4–4.1)	3.8(3.4–4.1)	3.7 (3.2–4.0)	**0.01**
Energy intake (kJ)	6322(5068–7870)	6261(5044–7825)	6617(5390–8121)	0.08	428(77–1045)	453(91–1057)	293 (36–841)	**0.02**
Blood Pressure (mmHg)								
Systolic	124.0(112.0–136.0)	123.0(112.0–135)	126.0(116.0–139.0)	0.11	138.0(128.0–151.0)	138.0(128.0–151.0)	140.0(127.0–152.0)	0.94
Diastolic	76.0(68.0–83.0)	76.0(68.0–83.0)	76.0(68.0–84.0)	0.36	83.0(76.0–89.0)	83.0(76.0–90.0)	81.0(73.0–88.0)	0.07
*Lifestyle factors*								
Smoking (current)	150(14.1%)	134(14.6%)	16(11.0%)	0.11	99(10.9%)	90(11.4%)	9 (7.4%)	0.20
Physically active	830(77.9%)	725(78.9%)	105(71.9%)	0.06	654(71.8%)	567(71.9%)	87 (71.3%)	0.90
Alcohol intake (g/d)	2.7(0.3–11.9)	3.1(0.3–12.0)	1.6(0.3–10.5)	0.13	12.1(2.1–28.7)	12.9(2.5–30.0)	8.0 (1.0–27.0)	**0.02**
Medication use								
Antidepressant	132(12.4%)	110(12.0%)	22(15.1%)	0.30	65(7.1%)	51(6.5%)	14 (11.5%)	**0.05**
Sedative	27(2.5%)	25(2.7%)	2 (1.4%)	0.57	10(1.1%)	10(100%)	0 (−)	0.21
Diabetic status	65(6.1%)	48(5.2%)	17(11.6%)	**<0.01**	43(4.7%)	37 (4.7%)	6 (4.9%)	0.91
Health status(current)				**0.03**				0.44
Excellent	165(15.5%)	146(13.7%)	19(1.8%)		119(13.1%)	109(12.0%)	10 (1.1%)	
Very good	455(42.7%)	401(37.7%)	54(5.1%)		398(43.7%)	344(37.8%)	54 (5.9%)	
Good	321(30.1%)	271(25.4%)	50(4.7%)		302(33.2%)	258(28.4%)	44 (4.8%)	
Fair	104(9.8%)	88(8.3%)	16(1.5%)		77(8.5%)	66 (7.3%)	11 (1.2%)	
Poor	20(1.9%)	13(1.2%)	7 (0.7%)		14(1.5%)	11 (1.2%)	3 (0.3%)	
Socioeconomic status (current)				0.20				0.79
Quintile 1(most disadvantaged)	169(15.9%)	140(13.2%)	29(2.7%)		149(16.4%)	127(13.9%)	22 (2.4%)	
Quintile 2	225(21.2%)	202(19.0%)	23(2.2%)		181(19.9%)	160(17.6%)	21 (2.3%)	
Quintile 3	242(22.8%)	204(19.2%)	38(3.6%)		175(19.2%)	153(16.8%)	22. (2.4%)	
Quintile 4	209(19.7%)	178(16.8%)	31(2.9%)		200(22.0%)	169(18.6%)	31 (3.4%)	
Quintile 5	217(20.4%)	192(18.1%)	25(2.4%)		206(22.61%)	180(19.8%)	26 (2.9%)	

Values are given as median (interquartile range), mean (±standard deviation) or n (%).

*EDS = ESS score ≥10.

BMI groups: Normal = BMI <25 kg/m^2^, Overweight = BMI ≥25–<30 kg/m^2^, Obese = BMI ≥30 kg/m^2^.

Those who reported EDS were more likely to be overweight or obese. Women who report EDS were more likely have a greater waist circumference, were more likely to be classified as centrally obese (waist circumference ≥88 cm), and had greater fat mass than those who were did not report EDS. No differences were detected between women with and without EDS in terms of %body fat, % BMC, %lean mass, energy intake, or blood pressure (diastolic and/or systolic).

Relationships between EDS and lifestyle factors are also shown in Table 1. Those with EDS were more likely report negative health status, and higher instances of ‘poor’ self-perceived health. Those with EDS were more likely to have diabetes than those who were not sleepy. No differences were found in terms of socioeconomic status, smoking status, medication use, physical activity or alcohol intake between those with and without EDS.

**Figure 1:**
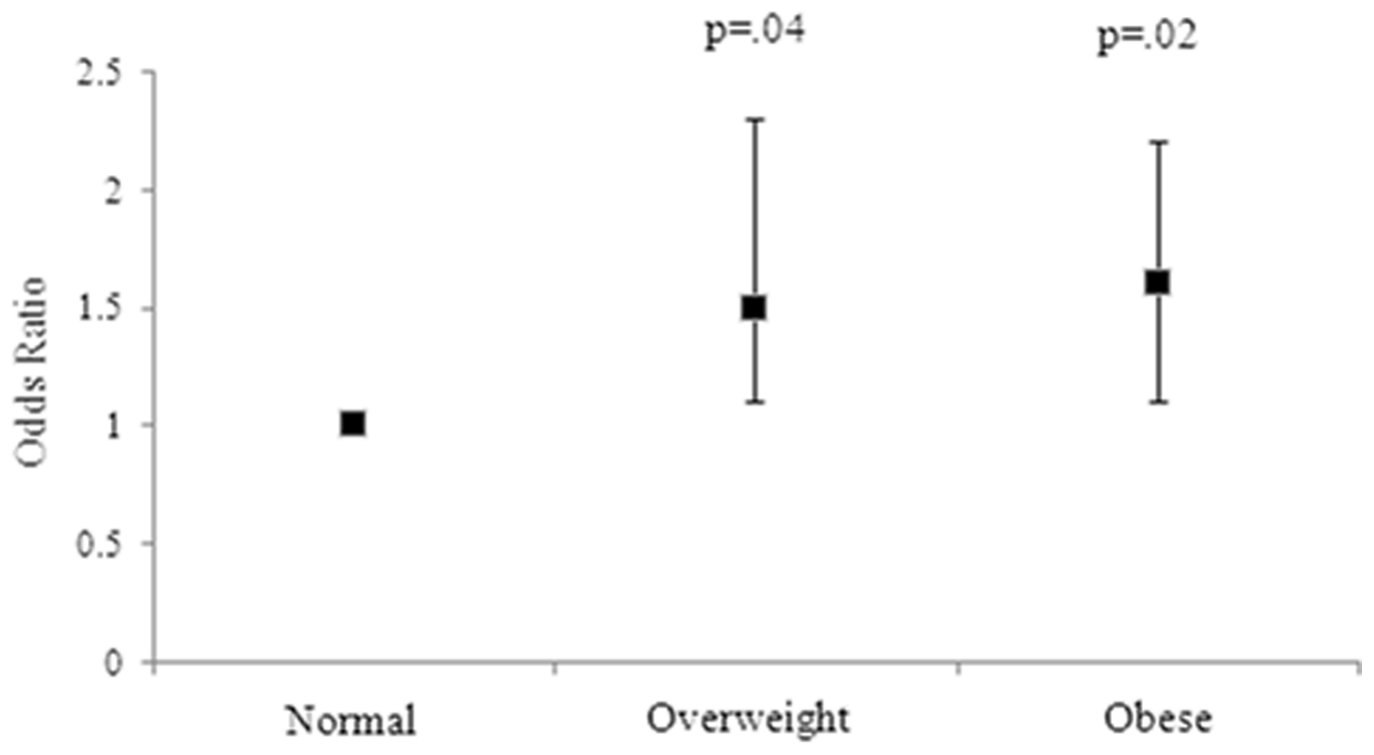
Adjusted odds ratios for women with EDS for BMI groups. Error bars represent the 95% CI. Group 1 (ideal BMI) is the reference group, with a broken line indicating the threshold of significance.

After adjusting for age, alcohol intake, antidepressant medication use and physical activity, EDS was associated with greater total waist circumference [Mean IQR: 93.6 (91.2–96.1) vs. 91.0 (89.5–92.6) cm, p = 0.03] and BMI [Mean IQR: 30.0 (29.0–31.1) vs. 29.1 (28.4–29.7) kg/m^2^, p = 0.07]. Compared to women of normal weight, having EDS was also associated with 1.5-fold increased odds of being overweight (adjusted OR = 1.5, 95%CI 1.0–2.3, p = 0.04), and 1.6-fold increased odds of being obese (adjusted OR = 1.6, 95%CI 1.1–2.3, p = 0.02) (Figure 1). These findings were not explained by the use of sedative medication, energy intake, or smoking status. A trend towards significance was noted for overall weight [Mean IQR: 78.6 (48.2–109.0) vs. 76.0 (74.3–77.8) kg, p = 0.07], and for those with a waist circumference ≥88 cm (adjusted OR = 1.4, 95%CI 1.0–2.0, p = 0.08). No differences in %body fat, %lean mass or fat mass were detected between those with and without EDS when assessed using multivariate modelling.

### Men

Subject characteristics are presented in Table 1. Of the 911 men included in this study, 122 (13.4%), were classified as having EDS. Men with EDS were more likely to be older and tended to be shorter in height than those men who did not report EDS. Overall, a considerable proportion of this sample of men met criteria for overweight (50.3%) and obesity (22.4%). Those men who reported EDS were more likely to have a larger overall waist circumference, and were more likely to be classified as centrally obese (waist circumference ≥102 cm). Those with EDS reported lower daily energy intake and %BMC than those who did not report EDS, and reported lower mean diastolic blood pressure. No differences were found in terms of overall weight, overweight and/or obesity based on BMI, %body fat, % lean mass, lean mass or fat mass between groups.

Differences between lifestyle factors among those men with and without EDS are presented in Table 1. Men with EDS were more likely to report lower daily alcohol intake and report higher rates of antidepressant medication use than those who were not sleepy. No differences were found with regard to smoking status, sedative use, diabetic status, health status, or socioeconomic status between those with and without EDS.

**Figure 2:**
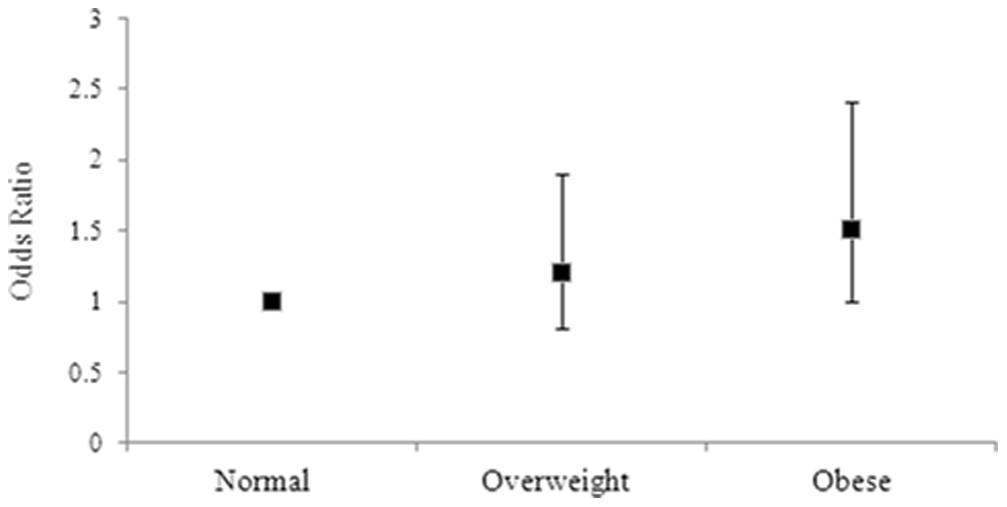
Adjusted odds ratios for men with EDS for BMI groups. Error bars represent the 95% CI. Group 1 (ideal BMI) is the reference group, with a broken line indicating the threshold of significance.

After adjusting for age, alcohol use, physical activity and smoking status, EDS was associated with greater BMI [Mean IQR: 28.6 (27.5–29.7) vs. 27.2 (26.9–28.6) kg/m^2^, p = 0.03]. These findings were not explained by the use of sedative or antidepressant medication use or energy intake. Compared to men of normal weight, having EDS was also associated with a 1.5-fold increased odds of being obese (adjusted OR = 1.5, 95%CI 1.0–2.4, p = 0.08) (Figure 2), independent of these factors. No differences in total weight, total waist circumference, being overweight, %body fat, % BMC or fat mass were found between those men with and without EDS.

## Discussion

This cross-sectional study identified a positive association between EDS and several measures of adiposity among Australian men and women. For women, after adjusting for medication and lifestyle factors, EDS was associated with greater total weight, waist circumference and BMI. Compared to women of normal weight, having EDS was also associated with 1.5-fold increased odds of being overweight and 1.6-fold increased odds of being obese (on BMI criteria). A trend towards significance was also noted for those women with EDS similarly having a waist circumference greater than 88 cm. For men, following adjustments, compared to men of normal weight, having EDS was associated with 1.5-fold increased odds of being obese, as well as greater BMI. These findings were not explained by the use of sedative or antidepressant medication use or energy intake.

A novel finding of this study was the relationship between EDS and several markers of adiposity, particularly among women, following the application of waist circumference [28]. To our knowledge, this is the first study to demonstrate an association between EDS and objectively measured viscerally located fat as waist circumference has previously been shown to be an effective measure of abdominal obesity, particularly when used in conjunction with BMI [29]. Women who reported EDS were more likely to have greater weight and BMI, and were more likely to be overweight or obese than women who were not sleepy. This finding is consistent with most, but not all of the existing literature [30]. In a population-based study of 5508 women residing in Sweden, participants who reported EDS were more likely to be classified as obese, as indicated by, and less likely to be within the ideal BMI range (defined as 20-<25 kg/m^2^) than women who were not sleepy [28]. This finding is similar to comparable research investigating EDS and BMI among population samples [16]. Bixler and colleagues (2005) found a robust relationship between EDS and BMI, demonstrating that the prevalence of EDS increased exponentially in those classified as overweight (average BMI = 28 kg/^2^). A similar finding was reported by both Vgontzas et al (1998) and Resta et al (2003). Notably, our research suggests that this association is comparably strong among both overweight and obese women. Clinically, this finding is important as it suggests the pervasiveness of these symptoms. Moreover, such findings address the need for greater emphasis on gender-specific treatment of EDS. Of the observed studies, a lack of specificity regarding BMI classifications among female populations [28] or the use of a combination of both male and female participants [16] means that current recommendations regarding female patients presenting with EDS is limited. Assessment of factors associated with increased adiposity where female patients present with symptoms of EDS may assist in streamlined treatment.

We demonstrated that men who reported EDS were more 1.5 times more likely to be classified as obese, and were more likely to report a greater overall BMI than those who were not sleepy. These findings are, in part, concordant with previous research, which has demonstrated significant associations between EDS and measure of obesity [16]. Compared to women, men with EDS reported less overall energy intake, and reported less alcohol use than those men who were not sleepy. Identification of adaptive lifestyle and health behaviours, may therefore, present as protective factors, however further examination is warranted in order to assess the magnitude of these associations.

Waist circumference has previously been cited as a risk factor for both obesity-related health risks such as sleep apnoea, metabolic disturbance and all-cause mortality [31], however, this finding is not universal [32]. We demonstrated that both men and women who reported EDS were more likely to report greater waist circumference. As waist measurements of ≥88 cm in women has been associated with increased risk for cardiovascular disease, diabetes and metabolic syndrome [33], further analysis is warranted to assess the impact of both the antecedent and impact of these factors within population samples, and the relationship to sleep heath. These findings were similarly represented among men when univariate models were applied; however this was not replicated following the application of multivariate regression modelling. Indeed, traditional measures of abdominal obesity have been shown to correlate poorly with sleep disturbances [34]. Therefore, assessment of upper abdominal obesity and measurements of neck circumference may be more effective in assessing the degree of sleep disturbances in these populations [11], however, this guideline is contentious [35]. More detailed study is required to assess the relationship between EDS and the relative distribution of visceral fat in men if more definitive conclusions are to be drawn.

The association between EDS and markers of adiposity within this sample may, in part, be mediated by increased inflammatory processes. Obesity is considered to represent a state of low-grade inflammation of white adipose tissue, as a result of chronic activation of the immune response [36]. Such processes increase the risk for subsequent development of diabetes mellitus via insulin resistance and reduction in glucose tolerance [36]. The inflammatory cytokine, Interleukin 6 (IL-6), has previously been shown to be a primary determinant for EDS [37], and serum IL-6 levels are associated with visceral adiposity [38]. Increased inflammatory responses have also been attributed to diseases often associated with obesity, such as diabetes. We demonstrated that women with EDS exhibited higher rates of diabetes than those who did not report these symptoms, which is consistent with previous research that demonstrated a link between sleepiness and the presence of underlying metabolic syndrome [39], and in line with studies suggesting that untreated EDS is an independent risk factor for the later development of diabetes among women [40]. However, we report that no differences were found between men in regard to diabetic status. It is acknowledged that information regarding diabetes within this sample was assessed via self-report. Moreover, blood pressure assessments were not routinely obtained at the same time each day, and therefore some diurnal variation may remain unaccounted for in these results. Thus, further data regarding the role of EDS in metabolic functioning may assist in clarity of results.

Increased prevalence of EDS among obese individuals have also been cited to result from underlying psychiatric illness [12]. Indeed, we have previously demonstrated that women with EDS are more likely to meet criteria for current or lifetime history of a depressive disorder [41]. As mental disorders were not examined for this study, it is possible that symptoms of EDS may, in part, by mediated by these underlying disorders. Research conducted by Williams et al (2009) reported that women with a lifetime history of psychiatric illness are more likely to meet criteria for adiposity than controls, therefore alluding to an association between body composition and mental health [42]. To our knowledge, however, no such comparison has been made between EDS and psychiatric disorders specifically, and thus, any interpretations at this time are premature.

No association was noted between EDS and measures of body composition as assessed by the whole body DXA scans for women or men, with the exception that men with EDS had lower %BMC than those who did not report EDS. As we did not have regional DXA assessment of the central area, we are unable to make inferences regarding central fat deposition. Further research is therefore needed to assess the association between EDS and the distribution of body fat.

Several methodological limitations must be identified when interpreting the findings of the current study. Given that the study was cross-sectional, no inferences can be made as to the directionality of the observed relationships between EDS and adiposity. Therefore, it is possible that the relationship may be bidirectional. Moreover, interpretation of the results may not generalizable to other populations or regions. Third, it is acknowledged that not all conditions that may affect EDS were assessed, including insomnia or obstructive sleep apnoea (OSA), which have been shown to be associated with EDS, particularly in clinical samples [24]. We did not explicitly assess the presence of underlying sleep disorders such as OSA, and therefore cannot exclude that this may have contributed to our findings. Despite this, research has shown a poor correlation between OSA and EDS in population-based cohorts [16] and we have included a comprehensive number of body composition assessments, which have been shown to correlate with disease severity. Nevertheless, to our knowledge, this is the first research to investigate the relationship between EDS and adiposity utilizing several anthropometric assessment measures in a population-based sample of men and women. Moreover, we assessed and accounted for a number of lifestyle and health factors, which are recognised as being implicated in EDS, therefore addressing limitations of past research.

We demonstrated that EDS is associated with increased measures of adiposity, particularly in women, independent of age, lifestyle factors and medication use. Given that both EDS and markers of adiposity constitute significant markers for disease, the findings of the study highlight the need to incorporate anthropometric measures in routine clinical assessment of patients.
